# Sex differences among elderly ACS patients undergoing percutaneous coronary intervention receiving Ticagrelor 60 mg vs. 90 mg

**DOI:** 10.1007/s11239-025-03206-y

**Published:** 2025-11-16

**Authors:** Raffaele Piccolo, Angelo Laino, Antonio Pio Vitale, Mario Enrico Canonico, Marisa Avvedimento, Fiorenzo Simonetti, Roberta Paolillo, Fabrizio Dal Piaz, Bruno Charlier, Alessandra Spinelli, Stefano Cristiano, Luigi Di Serafino, Plinio Cirillo, Giuseppe Gargiulo, Anna Franzone, Amelia Filippelli, Valeria Conti, Giovanni Esposito

**Affiliations:** 1 Department of Advanced Biomedical Sciences, University of Naples Federico II, Naples, Italy; 2 Department of Medicine, Surgery and Dentistry, Scuola Medica Salernitana, University of Salerno, Baronissi, SA Italy; 3 Clinical Pharmacology Unit, University Hospital San Giovanni di Dio e Ruggi d Aragona, Salerno, Italy

**Keywords:** Sex, Elderly, Acute Coronary Syndromes, Platelets, Ticagrelor

## Abstract

No sex-based data are available on the pharmacodynamic and pharmacokinetic profile of ticagrelor 60 mg twice daily as alternative to its standard dose during the early phase after acute coronary syndrome (ACS). This post-hoc study is a sex-based secondary analysis of the PLINY THE ELDER randomized, crossover trial (NCT04739384), which compared ticagrelor 60 vs. 90 mg in elderly patients with ACS undergoing percutaneous coronary intervention (PCI). The primary endpoint was the pre-dose P2Y_12_ reaction units (PRU) using the VerifyNow-P2Y_12_ (Accumetrics, San Diego, CA, USA) at 14 days after treatment with ticagrelor 60 or 90 mg twice daily. A total of 50 elderly patients with ACS was included in the study. Of these patients, 28 (56%) were males and 22 (44%) females. The two doses of ticagrelor had a comparable PRU in both males (pre-dose: LSM difference_60 vs. 90_ -7.00, 95%CI -25.3 to 11.3, *p* = 0.44; post-dose: LSM difference_60 vs. 90_ 3.90, 95%CI -10.6 to 18.5, *p* = 0.59) and females (pre-dose: LSM difference_60 vs. 90_ -0.89, 95%CI -20.3 to 18.5, *p* = 0.93; post-dose: LSM difference_60 vs. 90_ -1.10, 95%CI -16.6 to 14.3, *p* = 0.88), with no evidence of sex-based interaction (pre-dose: p for interaction = 0.88; post-dose: p for interaction = 0.65). Consistently, transmittance aggregometry and multiple electrode aggregometry showed a similar pharmacodynamic profile between the two doses of ticagrelor in both male and female patients. Plasma levels of ticagrelor were significantly lower using the reduced dose of ticagrelor as compared with the standard dose in both males (pre-dose: LSM difference_60 vs. 90_ -212, 95%CI -391 to -33.0, *p* < 0.002; post-dose: LSM difference_60 vs. 90_ -308, 95%CI -510 to -105, *p* = 0.004) and females (pre-dose: LSM difference_60 vs. 90_ -131, 95%CI -332 to 69.1, *p* = 0.19; post-dose: LSM difference_60 vs. 90_ -670, 95%CI -898 to -442, *p* < 0.001). Ticagrelor 60 mg and ticagrelor 90 twice daily yielded the same magnitude of platelet inhibition among elderly patients with ACS irrespective of sex.

**Graphical abstract:**

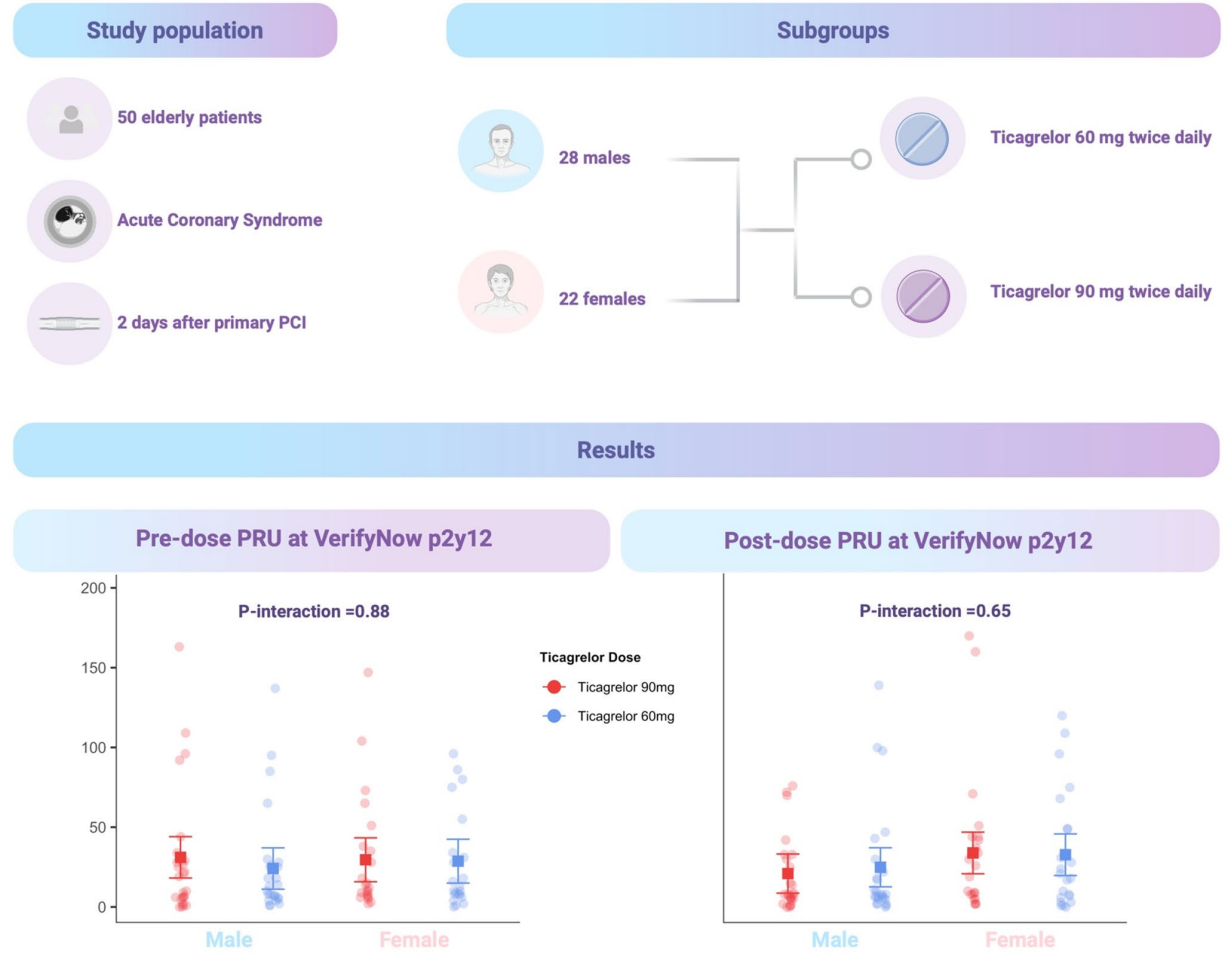

**Supplementary Information:**

The online version contains supplementary material available at 10.1007/s11239-025-03206-y.

## Introduction

 Dual antiplatelet therapy (DAPT), including aspirin and an oral P2Y_12_ receptor inhibitor, represents the standard of care for the prevention of recurrent ischemic events in patients with acute coronary syndrome (ACS) [1, 2]. However, DAPT is invariably associated with a heightened risk of bleeding [3], which may override its ischemic benefit in specific subsets [4, 5].

In this respect, identifying antithrombotic strategies able to reduce the incidence of bleeding events without affecting the ischemic risk is of clinical relevance to improve the net clinical benefit of DAPT [6, 7]. However, less-intensive antithrombotic strategies have been mainly investigated during the long-standing phase of ACS, whereas few data are available in the early phase post-ACS. In the PLINY THE ELDER (PLatelet Inhibition with two different doses of potent P2y12 inhibitors in THE ELDERly population) trial, we found a similar magnitude of platelet inhibition between ticagrelor 90 mg and 60 mg twice daily among elderly patients (≥ 75 years) during the early phase after ACS [8]. However, generalizability of study findings to female patients is uncertain given the sex-related differences in the biology of atherothrombosis [9, 10], potentially influencing the pharmacodynamic response to antiplatelet agents, including ticagrelor [11].

Herein, we reported a post-hoc analysis of the PLINY THE ELDER trial aiming to evaluate the pharmacodynamic and pharmacokinetic profile of ticagrelor 60 mg twice daily vs. ticagrelor 90 mg twice daily in male and female patients.

## Methods

### Study design and patients

The study design, inclusion and exclusion criteria of the PLINY THE ELDER trial have been previously described [8, 12]. Briefly, the study was an investigator-initiated, single-center, non-inferiority, open-label, two-by-two crossover, randomized trial, testing platelet inhibition with ticagrelor 60 mg twice daily compared with ticagrelor 90 mg twice daily in elderly patients with ACS undergoing PCI (ClinicalTrials.gov NCT04739384). Patients were eligible if they were aged 75 years or more, underwent successful PCI for non-ST-segment elevation ACS or ST-elevation myocardial infarction, and received a loading dose of ticagrelor of 180 mg. The principal exclusion criteria were indication to oral anticoagulant therapy, concomitant use of glycoprotein IIb/IIIa inhibitors or fibrinolytic agents, active bleeding, severe anemia, or chronic kidney disease at stage 4 or 5 (estimated glomerular filtration rate less than 30 mL/min/1.73m^2^). The crossover trial had 2-sequence and 2-period with patients randomized to either ticagrelor 60 mg twice daily (day 1–14) followed by ticagrelor 90 mg twice daily (day 15–28) or ticagrelor 90 mg twice daily (day 1–14) followed by ticagrelor 60 mg twice daily (day 15–28). A low dose aspirin (100 mg daily) was used in all patients. After the trial period, patients resumed their initial antiplatelet regimen if still indicated (ticagrelor 90 mg twice daily).

The study adhered to the ethical principles outlined in the Declaration of Helsinki, the specifications of the International Conference of Harmonization, and the guidelines of Good Clinical Practice. The study protocol was approved by the Italian Medicines Agency (EudraCT 2019–002391-13) and the Medical Ethics Committee of the University of Naples “Federico II”. All patients provided written informed consent.

### Randomization and masking

Randomization was allowed between 1 and 3 days after PCI and was conducted via a website (RedCap) using a computer-generated sequence with variable block sizes of 2 or 4. The sequence of block sizes was also randomly generated to further enforce concealment. Patients and the treating physicians were aware of group allocations, whereas personnel performing pharmacodynamic and pharmacokinetic testing was masked to the assigned treatment.

### Blood sampling

Blood sampling to evaluate adenosine diphosphate (ADP) and non-ADP platelet aggregation was performed at 3 time points: (a) time 1 (baseline): before randomization; (b) time 2 (crossover): 14 days after randomization, including 2 samples, before and 2 h after the last dose of the initial assigned treatment; (c) time 3 (end of study): 28 days after randomization, including 2 samples, before and 2 h after the last dose of the second assigned treatment.

Baseline venous blood collection was performed after successful PCI by dedicated nursing staff using a short venous catheter inserted into an arm vein. The first 2 to 4 mL of blood were discarded to avoid spontaneous platelet activation. All patients received a 250 mg bolus of aspirin administered by the emergency medical service, together with 5,000 IU of intravenous unfractionated heparin. During PCI, patients were treated with unfractionated heparin (at least 5,000 IU or an initial bolus of 70 IU/kg bodyweight) to maintain an activated clotting time >250 s. Venous blood was collected 1–3 days after PCI into citrate (0.129 mol/L) vacutainers for VerifyNow and light transmittance aggregometry (LTA), whereas samples for multiple electrode aggregometry (MEA) were drawn into hirudin tubes. Sample storage and processing were performed by trained personnel blinded to treatment allocation. Venous blood samples for both phase 1 and phase 2 were collected 12 h ± 2 h after the last maintenance dose of ticagrelor (pre-dose; the drug was taken by the patient the night before the post-visit blood collection) and 2 h after administration (post-dose; the drug was administered by dedicated nursing staff in hospital). All blood samples were maintained at room temperature during transport and analyzed by dedicated and trained personnel. Laboratory personnel used standardized criteria to assess each determination for validity for each assay type. For the VerifyNow, an electronic quality control was run on the day of blood analysis to verify instrument optics, pneumatics, and reagent mixing functions. For the MEA, the device temperature of the measurement system is controlled by the analyzer. The aggregation was measured for six minutes and the resulting two curves (measured by two independent sensor units) were displayed as area under the curve (AUC) and expressed in units (U). If Pearson’s correlation coefficient was < 0.98 or the difference of each curve from the mean was >20%, the analysis was repeated. Following the manufacturers’ recommendations, all samples were analyzed between 30 min and 3 h after venipuncture. Platelets aggregometry was performed at 37 °C following standard procedure [12, 13]. Conditions leading to invalidation of LTA samples included, but were not limited to, hemolysis, very low platelet counts in platelet-rich plasma (< 150,000/µL), and unstable baselines in LTA tracings indicative of sample temperature fluctuation or pre-agonist platelet activation.

### Pharmacodynamic assessment

Platelet function was assessed by using the VerifyNow, LTA, and MEA, each calibrated daily according to the manufacturer’s instructions.

The VerifyNow-P2Y_12_ (Accumetrics, San Diego, California) measures ADP-induced platelet agglutination as an increase in light transmittance and utilizes a proprietary algorithm to report values as P2Y_12_ reaction units (PRU), percent inhibition, and baseline value (BASE) for platelet function. In general, a higher PRU result reflects an increased P2Y_12_-mediated platelet reactivity, and, based on experts consensus, high platelet reactivity (HPR) is defined as PRU > 208. The VerifyNow Aspirin (CPT 85576) is a qualitative test for the detection of aspirin-induced platelet dysfunction. The test is reported in Aspirin Reaction Units (ARU). The therapeutic range for platelet function is 350–549 ARU while non-therapeutic range for platelet function is 550–700 ARU.

LTA uses a dual channel lumi-aggregometer (model 700; Chrono-Log, Havertown, PA). Platelet-rich plasma was obtained by whole blood sample centrifugation at 150 g for 15 min, and, after its extraction, platelet-poor plasma was obtained by re-centrifugation of blood tubes at 1500 g for 10 min. After ADP (5 and 20 µmol/L), acid arachidonic (1 µmol/L) and Thrombin receptor activating peptide (TRAP) (15 µmol/L) addition as a pro-aggregatory stimulus, platelet aggregation was monitored at 37 °C with constant stirring (1200 rpm) and measured as the increase in light transmission for 6 min. LTA results are reported as a percentage of maximum platelet aggregation (MPA) and HPR is defined as MPA > 59% (LTA 20 µmol/L ADP) and MPA > 46% (LTA 5 µmol/L ADP).

MEA is assessed in whole blood by the Multiplate analyzer (Roche-Dynabyte Medical, Munich, Germany). Platelet aggregation was measured after addition of agonists in whole blood. After dilution of 300 µL of hirudin-anticoagulated whole blood with 0.9% NaCl solution for 3 min at 37 °C, 20 µL of ADP test was added. For non-ADP-induced pathways, different tests were performed: aspirin and thrombin receptor-activating peptide. Platelet aggregation was recorded for 6 min and the mean values of 2 independent determinations were reported as AUC (area under curve) in arbitrary units. HPR is defined as AUC > 46 U.

### Pharmacokinetic assessment

Plasma levels of ticagrelor and its active metabolite AR-C124910XX were evaluated to determine the pharmacokinetic profile of ticagrelor 60 and 90 mg. Approximately 30% of ticagrelor-induced platelet inhibition derives from its active metabolite AR-C124910XX, generated through cytochrome P450 3A4, which is at least as potent at the P2Y_12_ receptor as ticagrelor. Hence, the pharmacokinetic profile was determined by measuring plasma concentrations of both ticagrelor and its metabolite using high performance liquid chromatography-tandem mass spectrometry.

### Study endpoints

The pharmacokinetic and pharmacodynamic outcome measures of the PLINY THE ELDER were investigated according to sex. The primary endpoint of PLINY THE ELDER was the pre-dose PRU using the VerifyNow-P2Y_12_ at 14 days after treatment with ticagrelor 60 or 90 mg twice daily. Secondary endpoints included HPR status by VerifyNow-P2Y_12_, ADP-induced platelet reactivity (and HPR status) measured by LTA and MEA, non-ADP-induced platelet reactivity by MEA, and plasma level of ticagrelor and its active metabolite AR-C124910XX.

### Statistical analysis

Details on statistical analysis have been published elsewhere [8, 12].

Participants characteristics were summarized using median (Q1-Q3) or frequency (percentage) as appropriate.

For all study endpoints, the statistical comparisons between groups were performed using linear mixed-effect models, fitting baseline value of the corresponding platelet function test, sequence of allocation (ticagrelor 60 mg twice daily followed by ticagrelor 90 mg twice or ticagrelor 90 mg twice daily followed by ticagrelor 60 mg twice daily), period (day 1–14 and day 15–28) and the interaction between treatment group (ticagrelor 60 mg vs. ticagrelor 90 mg) x sex (male vs. female) as fixed effects, and patient id as random effect to account for the time-repeated measurements. Patients with missing data were excluded from the analyses.

Least-squares means (LSM) in each treatment arm, derived from the mixed-effect models, were provided along with the 95% confidence interval (CI). A 2-tailed P value of < 0.05 was considered statistically significant.

All analyses were performed using R version 4.4.2 (R Foundation for Statistical Computing, Vienna, Austria).

## Results

### Study population

Out of 50 patients enrolled in the PLINY THE ELDER study, 28 were males (56%) and 22 were females (44%). As shown in Table 1, clinical characteristics were largely comparable between male and female patients, however some differences were present. The proportion of patients with diabetes (64% vs. 36%), previous myocardial infarction (36% vs. 9.1%), previous PCI (21 vs. 4.5%), previous CABG (11% vs. 0%), prior stroke or TIA (11% vs. 4.5%) and history of bleeding (7.1% vs. 0%) was higher in males as compared with females.

The culprit-lesion in males was more commonly located in the left circumflex artery (29% vs. 18%), whereas the left anterior descending artery (54% vs. 68%) was the prevalent culprit-site in females. The proportion of patients undergoing PCI for STEMI was similar between males and females (61% vs. 55%, respectively). Otherwise, procedural characteristics were similar between the two groups (Table 2).

### Measures of P2Y_12_-mediated platelet response in males and females

**Fig. 1:**
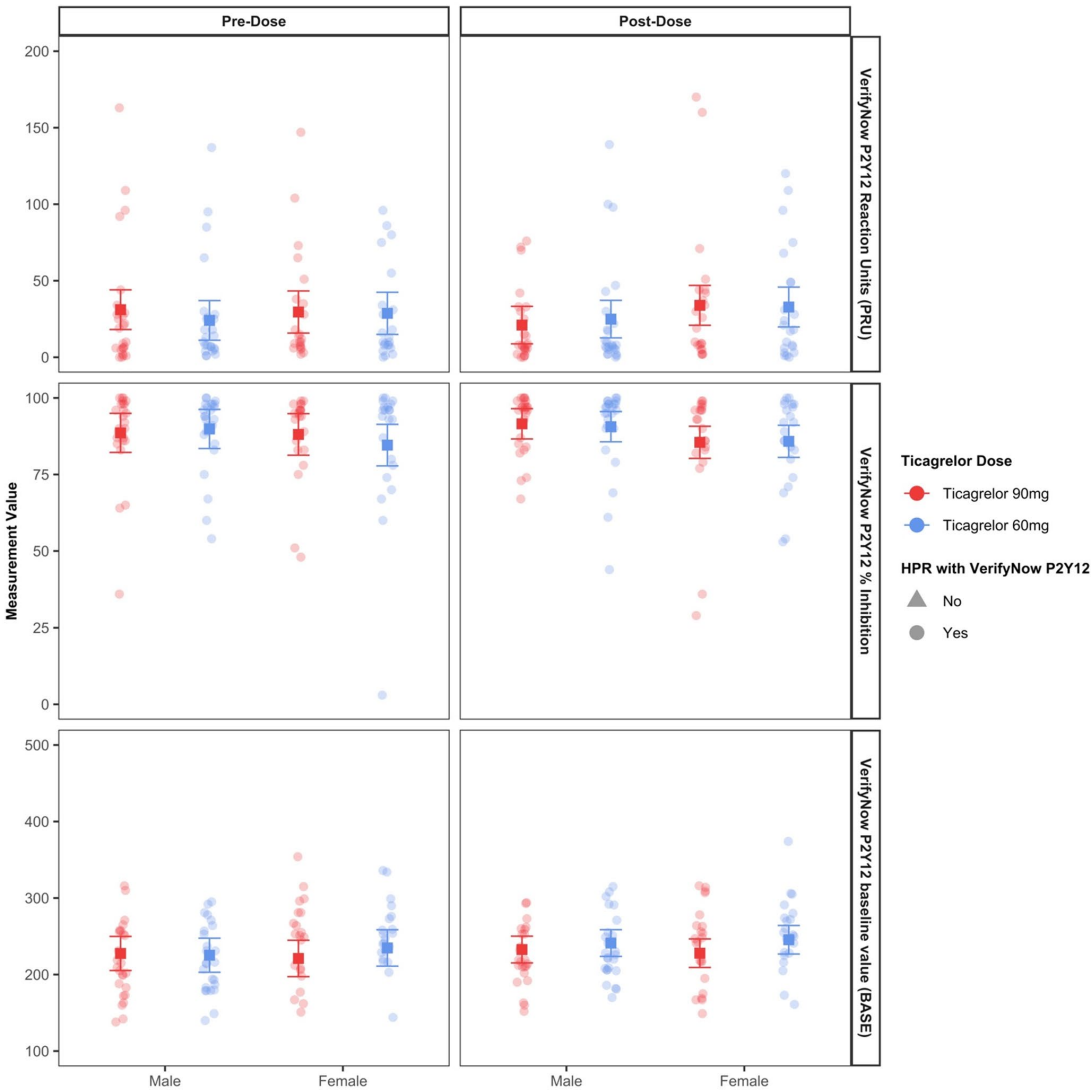
Measures of P2Y_12_-mediated platelet response with ticagrelor 60 mg compared to 90 mg twice daily in male and female patients using VerifyNow P2Y_12_ assay

**Fig. 2:**
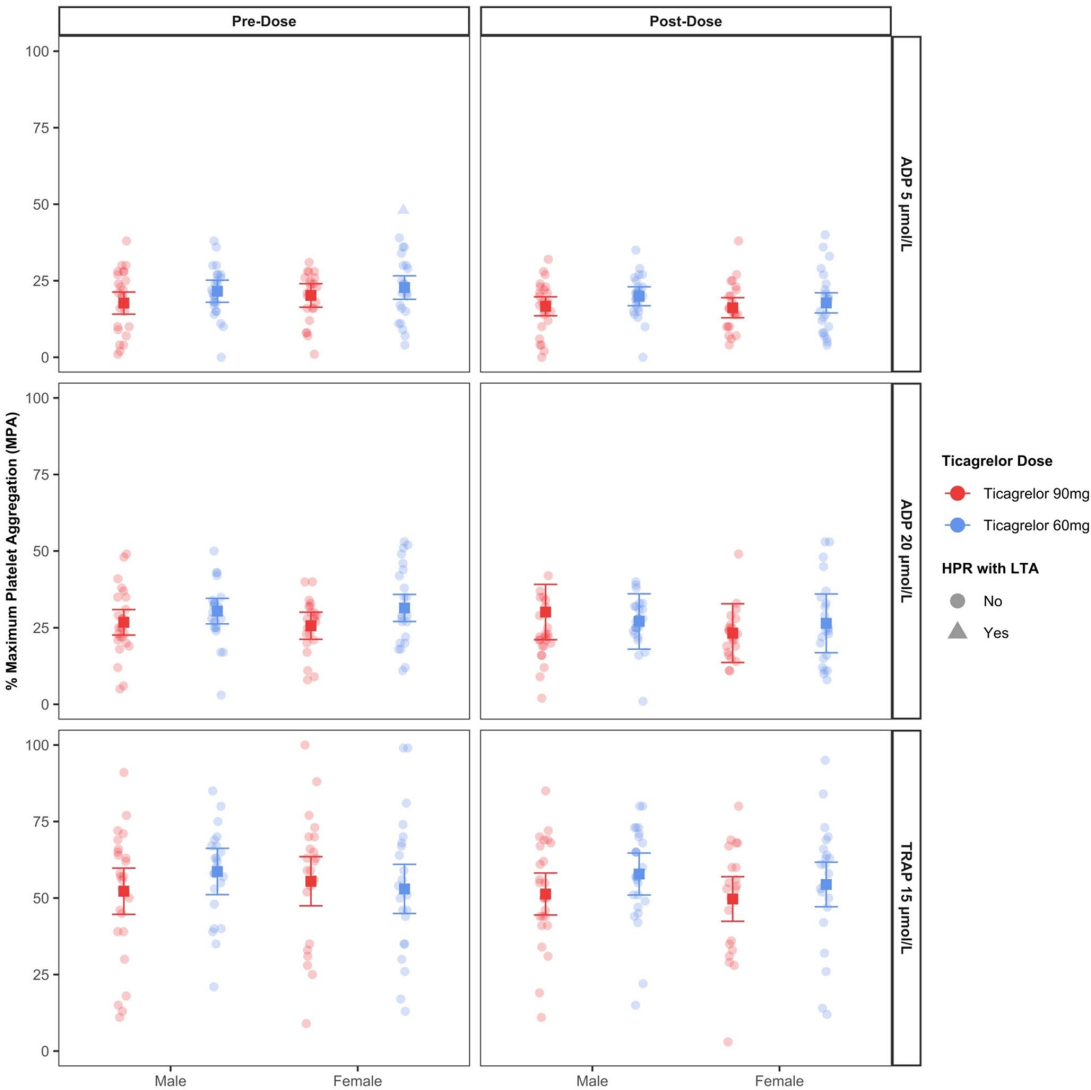
Measures of P2Y_12_-mediated platelet response with ticagrelor 60 mg compared to 90 mg twice daily in male and female patients using light transmittance aggregometry. *ADP* adenosine diphosphate; *LTA* light transmittance aggregometry

**Fig. 3:**
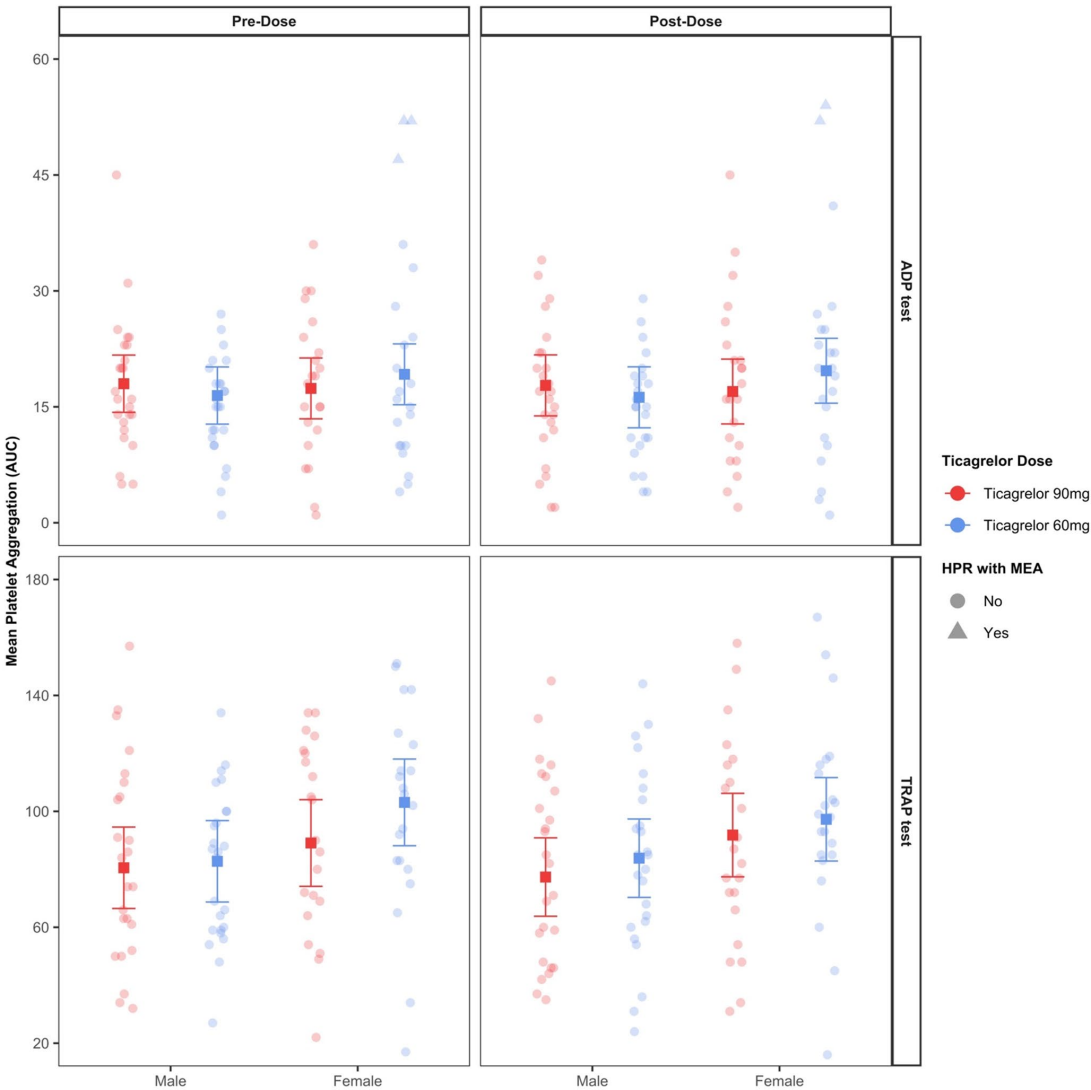
Measures of P2Y_12_-mediated platelet response with ticagrelor 60 mg compared to 90 mg twice daily in male and female patients using multiple electrode aggregometry. *ADP* adenosine diphosphate; *MEA* multiple electrode aggregometry

All measures of P2Y_12_-mediated platelet reactivity stratified by sex are shown in Figs. 1, 2 and 3.

Pre-dose PRU was comparable between ticagrelor 60 mg and ticagrelor 90 mg in both males (pre-dose: LSM difference_60 vs. 90_ −7.00, 95%CI −25.3 to 11.3, *p* = 0.44) and females (pre-dose: LSM difference_60 vs. 90_ −0.89, 95%CI −20.3 to 18.5, *p* = 0.93), with no significant interaction (pre-dose: p for interaction = 0.88) between randomized treatment and sex. Post-dose PRU was comparable between ticagrelor 60 mg and ticagrelor 90 mg in both males (post-dose: LSM difference_60 vs. 90_ 3.90, 95%CI −10.6 to 18.5, *p* = 0.59) and females (post-dose: LSM difference_60 vs. 90_ −1.10, 95%CI −16.6 to 14.3, *p* = 0.88) without significant interaction between treatment assignment and sex (post-dose: p for interaction = 0.65).

The VerifyNow P2Y_12_% inhibition at both pre-dose and post-dose was similar in the group randomized to ticagrelor 60 mg as compared with the group randomized to ticagrelor 90 mg in both males (pre-dose: LSM difference_60 vs. 90_ 1.30, 95%CI −7.75 to 10.3, *p* = 0.78; post-dose: LSM difference_60 vs. 90_ −0.90, 95%CI −7.04 to 5.18, *p* = 0.76) and females (pre-dose: LSM difference_60 vs. 90_ −3.50, 95%CI −13.1 to 6.10, *p* = 0.47; post-dose: LSM difference_60 vs. 90_ 0.30, 95%CI −6.16 to 6.82, *p* = 0.92), without significant interaction between ticagrelor dose and sex (pre-dose: p for interaction = 0.49, post-dose: p for interaction = 0.78).

Similarly, there was no interaction between ticagrelor dose in the early phase post-ACS and sex for the other measures of P2Y_12_-mediated platelet response (Table 3).

### High P2Y_12_-mediated platelet response in males and females

The proportion of male and female patients with HPR using the various measures of P2Y_12_-mediated platelet response are displayed in Figs. 1, 2 and 3 and eTable 1. Overall, patients randomized to ticagrelor 60 mg had a comparable proportion of HPR status with patients randomized to ticagrelor 90 mg in both males and females. Using the VerifyNow P2Y_12_ assays, none of the patients exhibited HPR-status at both pre-dose and post-dose assessment. The proportion of patients with HPR as measured in by LTA with ADP 5 µmol/L was similar in the group randomized to ticagrelor 60 mg vs. 90 mg in both males (pre-dose_60vs.90_: 0 patients [0%] vs. 0 patients [0%], post-dose_60vs.90_: 0 patients [0%] vs. 0 patients [0%]) and females (pre-dose_60vs.90_: 1 patient [5%] vs. 0 patients [0%], post-dose_60vs.90_: 0 patients [0%] vs. 0 patients [0%]). Similar results were found for HPR as measured by LTA with ADP 20 µmol/L. The proportion of patients with HPR as measured by MEA ADP test was similar between treatment groups in males (pre-dose_60vs.90_: 0 patients [0%] vs. 0 patients [0%], post-dose_60vs.90_: 0 patients [0%] vs. 0 patients [0%]), but higher in females treated with ticagrelor 60 mg vs. ticagrelor 90 mg (pre-dose_60vs.90_: 3 patients [14%] vs. 0 patients [0%], post-dose_60vs.90_: 2 patients [9%] vs. 0 patients [0%]).

### Measures of aspirin response in patients in males and females

Measures of aspirin response were comparable between ticagrelor 60 mg and ticagrelor 90 mg in both males and females at pre-dose assessment (eTable2, eFigure1).

Post-dose ARU was lower in patients treated with ticagrelor 60 mg regimen as compared with ticagrelor 90 mg regimen (post-dose: LSM difference_60 vs. 90_ −29.0, 95%CI −69.0 to 10.2, *p* = 0.14) in males. Conversely, in females, post-dose ARU was higher in patients treated with ticagrelor 60 mg vs. ticagrelor 90 mg (post-dose: LSM difference_60 vs. 90_ 48.0, 95%CI 5.66 to 89.8, *p* = 0.03), resulting in a significant interaction between treatment assignment and sex (post-dose: p for interaction = 0.01). Other measures of aspirin response were comparable between treatment groups in both males and females, although, a trend towards a lower response to aspirin in female patients assigned to the ticagrelor 60 mg regimen, was observed (eTable1, eFigure1).

### Pharmacokinetics results

Plasma levels of ticagrelor were significantly lower in ticagrelor 60 mg regimen compared to ticagrelor 90 mg regimen in both males (pre-dose: LSM difference_60 vs. 90_ −212, 95%CI −391 to −33.0, *p* < 0.002; post-dose: LSM difference_60 vs. 90_ −308, 95%CI −510 to −105, *p* = 0.004) and females (pre-dose: LSM difference_60 vs. 90_ −131, 95%CI −332 to 69.1, *p* = 0.19; post-dose: LSM difference_60 vs. 90_ −670, 95%CI −898 to −442, *p* < 0.001) (eTable3). Similarly, plasma levels of the ticagrelor metabolite AR-C124910XX were significantly lower in patients treated with ticagrelor 60 mg as compared with those treated with ticagrelor 90 mg in both males (pre-dose: LSM difference_60 vs. 90_ −162, 95%CI −250 to −73.2, *p* < 0.001; post-dose: LSM difference_60 vs. 90_ −156, 95%CI −247 to −63.7, *p* < 0.001) and females (pre-dose: LSM difference_60 vs. 90_ −97.0, 95%CI −196 to 2.14, *p* = 0.06; post-dose: LSM difference_60 vs. 90_ −244, 95%CI −348 to −141, *p* < 0.001) (eTable3).

## Discussion

In the present post-hoc analysis of the PLINY THE ELDER trial, we investigated the sex-based differences in the platelet response between a standard and low dose of ticagrelor in elderly patients with ACS undergoing PCI. Our principal findings are as follows:

1) Ticagrelor 60 mg twice daily provided a similar degree of platelet inhibition compared with ticagrelor 90 mg twice daily in both male and female patients;

2) In both males and females, the comparable effect on platelet inhibition was achieved despite the group randomized to ticagrelor 60 mg had lower plasma concentrations of ticagrelor and its active metabolite as compared with the group randomized to ticagrelor 90 mg;

3) Female patients treated with ticagrelor 60 mg showed a reduced response to aspirin as compared with those treated with ticagrelor 90 mg, differently, in male patients the aspirin response was comparable between treatment groups.

A substantial body of evidence indicates that, compared with males, females exhibit increased baseline platelet reactivity, which may influence their clinical response to various antiplatelet [14–18] therapies. Specifically, females have shown a reduced pharmacological response to aspirin and clopidogrel relative to males, as demonstrated in multiple studies over the years [15, 19–21]. In contrast, platelet reactivity appears similar between males and females treated with DAPT [18]. To date, three randomized trials have documented that ticagrelor 60 mg provides a level of platelet inhibition comparable to that of ticagrelor [8, 22, 23] 90 mg. Given the documented sex-related differences in platelet reactivity using antiplatelet drugs, it was essential to assess the extent to which these findings apply across sexes. Our study showed that ticagrelor 60 mg and ticagrelor 90 mg achieve a comparable magnitude of platelet inhibition in both male and female patients during the early phase post-ACS, with a median time from PCI to randomization of 2 days. These observations represent a central finding of our analysis and carry several important clinical implications.

First, our results reinforce the findings of the only study to date that has provided sex-based data for the comparison between ticagrelor 60 mg and ticagrelor 90 mg in patients with coronary artery disease. In the PEGASUS-TIMI 54 (Prevention of Cardiovascular Events in Patients With Prior Heart Attack Using Ticagrelor Compared to Placebo on a Background of Aspirin–Thrombolysis In Myocardial Infarction 54) trial, both ticagrelor 60 mg and ticagrelor 90 mg had a similar safety and efficacy in comparison to placebo during the longstanding phase of ACS, with no heterogeneity between randomization and sex [24]. Consistently, the pharmacodynamic substudy of PEGASUS-TIMI 54 trial found similar platelet inhibition with both ticagrelor doses [22]. However, the authors did not report data stratified by sex, limiting the generalizability of the pharmacodynamic results to female patients and underscoring the persistent underreporting of sex-specific outcomes in cardiovascular research. Our findings align with the clinical observations of the PEGASUS-TIMI 54 and extend its pharmacological substudy by revealing a comparable platelet inhibition achieved with the two ticagrelor doses in both male and female patients.

Second, our study is the first providing the mechanistic basis to evaluate in a randomized clinical trial a ticagrelor 60 mg-based antithrombotic strategy during the early phase post-ACS with the aim of optimizing the balance between ischemic and bleeding risks. This shortcoming can be mitigated weighing the benefits and risks for the individual patient, tailoring individually the more appropriate antithrombotic strategy [25]. In this context, our findings, demonstrating that there are no sex-related biological differences in platelet reactivity of patients treated with ticagrelor, support the rationale for testing a lower-dose ticagrelor strategy from a clinical perspective.

The observation of a sex-based interaction in the measures of aspirin response with ticagrelor 60 mg vs. ticagrelor 90 mg deserves attention. This result was supported by the VerifyNow Aspirin, however, with LTA and MEA we did not find an interaction between treatment allocation and sex. In this regard, it should be noted that discrepancies between point-of-care tests of aspirin response have already been described [26]. We are not able to provide a mechanistic explanation for this finding, which should be carefully interpreted. In healthy volunteers, aspirin had no influence on platelet aggregation in the presence of strong blockage of P2Y_12_ receptor [27]. However, other studies suggested that this relationship is dependent on the concentration of the P2Y_12_ receptor-inhibitor [28]. Whether these discrepancies may be enhanced by sex-based differences remains hypothesis generating in view of the high number of statistical tests performed in this study, and the lack of correction for multiplicity.

The results of this study must be interpreted in view of the following limitations. First, this was not a prespecified analysis, therefore, it has all the potential shortcomings of post-hoc studies. Second, randomization was not stratified by sex, therefore, our results may be partly attributable to baseline characteristics. Third, although there is general agreement that individuals over 75 years can be defined as “elderly”, ageing is a continuous process, and the cut-off of 75 years remains arbitrary. Fourth, discrepancies were observed among platelet function test results at the pre-dose evaluation. Specifically, in male patients, treatment with ticagrelor 60 mg was associated with reduced platelet reactivity according to the VerifyNow P2Y_12_ and MEA assays, whereas the opposite trend was observed with LTA, which showed lower platelet reactivity in the higher-dose group. Such inconsistencies are common with point-of-care assay, as already previously described, and may be attributed to differences in the underlying principles of the respective tests (use of whole blood vs. platelet rich plasma, the presence of different types of anticoagulant in the withdrawal tube, or detection methods), as well as to patient characteristics or concomitant medications use [26, 29–32]. The dynamic range of VerifyNow P2Y_12_ has been shown to be narrower when compared to that of LTA in several studies [33, 34]. This aspect may have contributed to the discrepancies between these assays observed in our analyses. Whether such differences are more pronounced in males has not been described previously, and given the limited sample size of our study, this finding should be regarded as exploratory. A major limitation in evaluating these inconsistencies, was the absence of data for key laboratory parameters at pre-dose assessment. Variables such as hemoglobin, creatinine and platelet count have been showed to influence the correlation between VerifyNow P2Y_12_ or MEA with LTA. These parameters were not collected before pre-dose evaluation and therefore, we were unable to explore their potential impact on platelet function tests results. For example, differences in hemoglobin levels may partly explain the poor correlation between whole-blood and platelet–rich–plasma–based assays, since low hematocrit can increase light transmission and platelet reactivity in both VerifyNow P2Y_12_ and MEA [35]. Furthermore, some confounding might have arisen from some aspects of concomitant medication use, which was not controlled by our design. For example, we were unable to control for the timing of aspirin administration before pre-dose assessment and variation in this covariate may partly account for the discrepancies observed across platelet function tests considered its lowering effect on LTA measured platelet reactivity [36]. Despite such limitations, it should be recognized that platelet reactivity mean values and ranges observed in our analyses were consistent with those reported in prior studies [23, 37, 38]. Moreover, these differences were not clinically meaningful, since all results in male patients remained well below the threshold for high platelet reactivity.

In conclusion, this post-hoc analysis of the PLINY THE ELDER trial showed that ticagrelor 60 mg twice daily provides a similar magnitude of platelet inhibition compared with ticagrelor 90 mg twice daily in both male and female patients despite lower drug concentrations observed with ticagrelor 60 mg twice daily.

**Table 1:** Baseline clinical characteristics of the PLINY THE ELDER participants

	Male *N* = 28^1^	Female *N* = 22^1^
Age	79 (77, 81)	80 (76, 83)
Body mass index, kg/m^2	27.7 (24.9, 30.1)	26.5 (23.1, 30.1)
Smoking	5 (18%)	4 (18%)
Hypertension	23 (82%)	19 (86%)
Dyslipidaemia	20 (71%)	14 (64%)
Diabetes	18 (64%)	8 (36%)
Congestive heart failure	4 (14%)	2 (9.1%)
Peripheral artery disease	0 (0%)	1 (4.5%)
Previous myocardial infarction	10 (36%)	2 (9.1%)
Previous PCI	6 (21%)	1 (4.5%)
Previous CABG	3 (11%)	0 (0%)
Prior stroke or TIA	3 (11%)	1 (4.5%)
History of bleeding	2 (7.1%)	0 (0%)
Chronic obstructive lung disease	5 (18%)	3 (14%)
Haemoglobin, (g/dL)	13.10 (12.00, 14.20)	11.75 (11.10, 13.60)
Platelet count, x 10^3^/µL	184 (162, 220)	260 (217, 326)
White blood cell count, x 10^3^/µL	9.15 (7.86, 10.63)	10.32 (7.23, 13.90)
eGFR, mL/min/1.73m^2^	49 (37, 59)	56 (36, 72)

^1^n (%); Median (Q1, Q3)

CABG: coronary artery bypass graft; eGFR: estimated glomerular filtration; PCI: percutaneous coronary intervention; TIA: transient ischemic attack

**Table 2:** Procedural characteristics of the PLINY THE ELDER participants

Characteristic	Male *N* = 28^1^	Female *N* = 22^1^
*Indication for revascularization*		
STEMI	17 (61%)	12 (55%)
N-STEMI	11 (39%)	8 (36%)
Unstable Angina	0 (0%)	2 (9.1%)
*Artery*		
Left main artery	1 (3.6%)	0 (0%)
Left anterior descending artery	15 (54%)	15 (68%)
Left circumflex artery	8 (29%)	4 (18%)
Right coronary artery	4 (14%)	3 (14%)
*Arterial access site*		
Femoral	5 (18%)	0 (0%)
Radial	23 (82%)	22 (100%)
Thrombotic lesion	10 (36%)	9 (41%)
Any bifurcation	4 (14%)	4 (18%)
*Number of implanted stents*		
1	20 (74%)	16 (76%)
2	7 (26%)	5 (24%)

^1^n (%); Median (Q1, Q3)

N-STEMI: non-ST-elevation myocardial infarction; STEMI: ST-elevation myocardial infarction

**Table 3:** Pharmacodynamic profile of Ticagrelor 60 mg vs. 90 mg twice daily stratified by sex

	Male *N* = 28				Female *N* = 22				*p*-interaction^2^
	LSM		LSM difference	*p*-value^1^	LSM		LSM difference	*p*-value^1^	
	Ticagrelor 60 mg	Ticagrelor 90 mg			Ticagrelor 60 mg	Ticagrelor 90 mg			
Pre-dose assessment (before the last dose of ticagrelor)									
*VerifyNow P2Y*_12_									
P2Y_12_ reaction units (PRU)	24.1 (11.1, 37.0)	31.1 (18.1, 44.0)	−7.00 (−25.3, 11.3)	0.44	28.7 (15.0, 42.5)	29.6 (15.8, 43.3)	−0.89 (−20.3, 18.5)	0.93	0.88
% inhibition	89.9 (83.5, 96.3)	88.6 (82.2, 95.0)	1.30 (−7.75, 10.3)	0.78	84.6 (77.8, 91.4)	88.1 (81.3, 94.9)	−3.50 (−13.1, 6.10)	0.47	0.49
Baseline value (BASE)	225 (203, 248)	228 (205, 250)	−3.00 (−24.5, 19.8)	0.83	235 (211, 259)	221 (197, 245)	14.0 (−9.80, 37.2)	0.25	0.34
*Light transmittance aggregometry (LTA)*									
% MPA with ADP 5 µmol/L	21.6 (18.0, 25.2)	17.7 (14.1, 21.3)	3.90 (−1.23, 8.99)	0.13	22.8 (18.9 26.6)	20.2 (16.4, 24.0)	2.60 (−2.85, 8.00)	0.34	0.74
% MPA with ADP 20 µmol/L	30.4 (26.3, 34.6)	26.8 (22.6, 31.0)	3.60 (−1.94, 9.26)	0.20	31.5 (27.1, 35.9)	25.7 (21.2, 30.1)	5.80 (−0.14, 11.8)	0.06	0.61
% MPA with TRAP 15 µmol/L	58.7 (51.1, 66.2)	52.2 (44.7, 59.8)	6.50 (−2.92, 15.8)	0.17	53.0 (45.0, 61.0)	55.5 (47.5, 63.5)	−2.50 (−12.5, 7.45)	0.62	0.21
*Multiple electrode aggregometry (MEA)* ^3^									
Mean platelet aggregation with ADP test	16.5 (12,8, 20.2)	18.0 (14.3, 21.7)	−1.50 (−6.34, 3.28)	0.53	19.2 (15.3, 23.1)	17.4 (13.5, 21.3)	1.80 (−3.29, 6.93)	0.48	0.36
Mean platelet aggregation with TRAP test	82.8 (68.7, 96.8)	80.5 (66.5, 94.6)	2.30 (−13.7, 18.1)	0.78	103 (88.1, 118)	89.1 (74.1, 104)	13.9 (−2.85, 30.9)	0.10	0.33
Post-dose assessment (2 h after the last dose of ticagrelor)									
*VerifyNow P2Y*_12_ assay									
P2Y_12_ reaction units (PRU)	24.9 (12.7, 37.2)	21.0 (8.76, 33.3)	3.90 (−10.6, 18.5)	0.59	32.8 (19.8, 45.8)	33.9 (20.9, 47.0)	−1.10 (−16.6, 14.3)	0.88	0.65
% inhibition	90.6 (85.7, 95.6)	91.5 (86.6, 96.5)	−0.90 (−7.04, 5.18)	0.76	85.8 (80.6, 91.1)	85.5 (80.3, 90.7)	0.30 (−6.16, 6.82)	0.92	0.78
Baseline value (BASE)	241 (224, 259)	233 (215, 250)	8.00 (−5.02, 22.0)	0.21	246 (227, 264)	228 (209, 247)	18.0 (3.20, 31.59)	0.02	0.38
*Light transmittance aggregometry (LTA)*									
% MPA with ADP 5 µmol/L	19.9 (16.8, 23.0)	16.7 (13.6, 19.7)	3.20 (−0.60, 7.15)	0.10	17.8 (14.5, 21.1)	16.2 (12.9, 19.5)	1.60 (− 2.54, 5.69)	0.44	0.56
% MPA with ADP 20 µmol/L	27.1 (18.0, 36.1)	30.1 (21.1, 39.2)	−3.00 (−15.40, 9.27)	0.62	26.4 (16.8, 36.0)	23.3 (13.7, 32.9)	3.10 (−9.91, 16.3)	0.63	0.50
% MPA with TRAP 15 µmol/L	57.8 (51.0, 64.7)	51.3 (44.5, 58.2)	6.50 (−0.72, 13.8)	0.08	54.4 (47.1, 61.7)	49.7 (42.4, 57.0)	4.70 (−2.96, 12.4)	0.22	0.74
*Multiple electrode aggregometry (MEA)* ^3^									
Mean platelet aggregation with ADP test	16.2 (12.3, 20.2)	17.8 (13.8, 21.7)	−1.60 (−6.33, 3.25)	0.52	19.7 (15.5, 23.9)	17.0 (12.8, 21.2)	2.70 (−2.40, 7.78)	0.29	0.25
Mean platelet aggregation with TRAP test	83.8 (70.3, 97.4)	77.3 (63.8, 90.9)	6.50 (−8.03, 21.0)	0.37	97.3 (82.9, 112)	91.8 (77.4, 106)	5.50 (−9.99, 20.9)	0.48	0.92

Values are least square means (95% CI). ADP: adenosine diphosphate; MPA: maximum platelet aggregation; TRAP: thrombin receptor activating peptide

^1^P-value for comparison between treatment

^2^P-value for interaction of ticagrelor 60 mg and ticagrelor 90 mg across diabetes groups

^3^Mean platelet aggregation is reported as area under curve (AUC)

## Supplementary Information

Below is the link to the electronic supplementary material.

Supplementary Material 1

## Data Availability

Requests for data sharing should be sent to the corresponding author a: Raffaele.Piccolo@unina.it.

## Declarations

### Copyright/license for publication

The Corresponding Author has the right to grant on behalf of all authors and does grant on behalf of all authors, a worldwide license to the Publishers and its licensees in perpetuity, in all forms, formats and media (whether known now or created in the future), to (i) publish, reproduce, distribute, display and store the Contribution, (ii) translate the Contribution into other languages, create adaptations, reprints, include within collections and create summaries, extracts and/or, abstracts of the Contribution, (iii) create any other derivative work(s) based on the Contribution, (iv) to exploit all subsidiary rights in the Contribution, (v) the inclusion of electronic links from the Contribution to third party material where-ever it may be located; and, (vi) license any third party to do any or all of the above.

### Guarantor information

RP is the guarantor for this work and accepts full responsibility for the conduct of the study, had access to the data, and controlled the decision to publish. The corresponding author (RP) attests that all listed authors meet authorship criteria and that no others meeting the criteria have been omitted.

### Transparency statement

The manuscript’s guarantor (RP) affirms that the manuscript is an honest, accurate, and transparent account of the study being reported; that no important aspects of the study have been omitted; and that any discrepancies from the study as originally planned (and, if relevant, registered) have been explained.
