# Supplementary material for: Sex differences among elderly ACS patients undergoing percutaneous coronary intervention receiving Ticagrelor 60 mg vs. 90 mg
Source: J Thromb Thrombolysis. 2025 Nov 16;59(3):551–63. doi: 10.1007/s11239-025-03206-y (PMC13246881; doi:10.1007/s11239-025-03206-y)

**Supplementary data legend**

**eTable 1**. Proportion of male and female patients with high P2Y_12_-mediated platelet reactivity.

**eTable 2**. Pharmacodynamic profile of aspirin in male and female patients.

**eTable 3**. Pharmacokinetic profile of ticagrelor 60 mg compared to 90 mg twice daily in male and female patients.

**eFigure 1**. Measures of aspirin response with ticagrelor 60 mg compared to 90 mg twice daily in male and female patients.

**eTable 1**.

|  | **Male** N = 28^1^ | | **Female**  N = 22^1^ | |
| --- | --- | --- | --- | --- |
|  | Ticagrelor  60 mg | Ticagrelor  90 mg | Ticagrelor  60 mg | Ticagrelor  90 mg |
| **Pre-dose assessment**  (before the last dose of ticagrelor) |  |  |  |  |
| PRU > 208 | 0  (0) | 0  (0) | 0  (0) | 0  (0) |
| % MPA with ADP 5 μmol/L > 46% | 0  (0) | 0  (0) | 1  (5) | 0  (0) |
| % MPA with ADP 20 μmol/L > 59% | 0  (0) | 0  (0) | 0  (0) | 0  (0) |
| Mean platelet aggregation with ADP test > 46^2^ | 0  (0) | 0  (0) | 3  (14) | 0  (0) |
| **Post-dose assessment**  (2h after the last dose of ticagrelor) |  |  |  |  |
| PRU > 208 | 0  (0) | 0  (0) | 0  (0) | 0  (0) |
| % MPA with ADP 5 μmol/L > 46% | 0  (0) | 0  (0) | 0  (0) | 0  (0) |
| % MPA with ADP 20 μmol/L > 59% | 0  (0) | 1  (4) | 0  (0) | 0  (0) |
| Mean platelet aggregation with ADP test > 46^2^ | 0  (0) | 0  (0) | 2  (9) | 0  (0) |

^1^n (%); ^2^Mean platelet aggregation is reported as area under curve (AUC).

**eTable2**.

|  | **Male** N = 28 | | | | **Female**  N = 22 | | | | **p-interaction^2^** |
| --- | --- | --- | --- | --- | --- | --- | --- | --- | --- |
|  | **LSM** | | **LSM**  **difference** | **p-value^1^** | **LSM** | | **LSM**  **difference** | **p-value^1^** |  |
|  | Ticagrelor  60 mg | Ticagrelor  90 mg |  |  | Ticagrelor  60 mg | Ticagrelor  90 mg |  |  |  |
| **Pre-dose assessment**  (before the last dose of ticagrelor) |  |  |  |  |  |  |  |  |  |
| **VerifyNow aspirin:** |  |  |  |  |  |  |  |  |  |
| Aspirin reaction units (ARU) | 420  (378, 463) | 440  (397, 482) | -20.0  (-.77.9, 39.2) | 0.51 | 428  (383, 473) | 420  (375, 465) | 8.00  (-54.0, 70.3) | 0.80 | 0.53 |
| **Light transmittance aggregometry (LTA):** |  |  |  |  |  |  |  |  |  |
| % MPA with arachidonic acid 1 μmol/L | 3.58  (0.32, 6.85) | 2.49  (-0.77, 5.76) | 1.09  (-3.00, 5.18) | 0.59 | 6.73  (3.26, 10.2) | 4.66  (1.18, 8.13) | 2.07  (-2.27, 6.42) | 0.34 | 0.75 |
| **Multiple electrode aggregometry (MEA)^3^:** |  |  |  |  |  |  |  |  |  |
| Mean platelet aggregation with ASPI test | 11.6  (8.08, 15.6) | 12.6  (8.89, 16.4) | -1.00  (-5.39, 3.78) | 0.72 | 12.4  (8.39, 16.3) | 10.8  (6.85, 14.8) | 1.60  (-3.33, 6.41) | 0.53 | 0.50 |
| **Post-dose assessment**  (2h after the last dose of ticagrelor) |  |  |  |  |  |  |  |  |  |
| **VerifyNow aspirin assay:** |  |  |  |  |  |  |  |  |  |
| Aspirin reaction units (ARU) | 426  (393, 459) | 455  (422, 488) | -29.0  (-69.0, 10.2) | 0.14 | 461  (426, 496) | 413  (378, 448) | 48.0  (5.66, 89.8) | 0.03 | 0.01 |
| **Light transmittance aggregometry (LTA):** |  |  |  |  |  |  |  |  |  |
| % MPA with arachidonic acid 1 μmol/L | 3.06  (-0.91, 7.02) | 3.12  (-0.84, 7.08) | -0.06  (-5.23, 5.10) | 0.98 | 8.19  (3.99, 12.4) | 4.04  (-0.17, 8.26) | 4.15  (-1.33, 9.64) | 0.13 | 0.28 |
| **Multiple electrode aggregometry (MEA)^3^:** |  |  |  |  |  |  |  |  |  |
| Mean platelet aggregation with ASPI test | 10.7  (6.70, 14.6) | 11.7  (7.70, 15.6) | -1.00  (-6.53, 4.53) | 0.72 | 14.2  (9.96, 18.4) | 12.6  (8.37, 16.8) | 1.60  (-4.29, 7.46) | 0.59 | 0.54 |

Values are least square means (95% CI). ADP: adenosine diphosphate; MEA: multiple electrode aggregometry; MPA: maximum platelet aggregation; LTA: light transmittance aggregometry. TRAP: thrombin receptor activating peptide.

^1^P-value for comparison between treatment.

^2^P-value for interaction of ticagrelor 60 mg and ticagrelor 90 mg across diabetes groups.

^3^Mean platelet aggregation is reported as area under curve (AUC).

**eTable3**.

|  | **Male** N = 28 | | | | **Female**  N = 22 | | | |
| --- | --- | --- | --- | --- | --- | --- | --- | --- |
|  | **LSM** | | **LSM**  **difference** | **p-value^1^** | **LSM** | | **LSM**  **difference** | **p-value^1^** |
|  | Ticagrelor  60 mg | Ticagrelor  90 mg |  |  | Ticagrelor  60 mg | Ticagrelor  90 mg |  |  |
| **Pre-dose assessment**  (before the last dose of ticagrelor) |  |  |  |  |  |  |  |  |
| Ticagrelor (ng/mL) | 346  (206-486) | 558  (418-697) | -212  (-391, -33.0) | 0.002 | 447  (290-603) | 578  (421-735) | -131  (-332, 69.1) | 0.19 |
| AR-C124910XX (ng/mL) | 229  (155-304) | 391  (316-465) | -162  (-250, -73.2) | < 0.001 | 290  (206-373) | 387  (303-470) | -97.0  (-196, 2.14) | 0.06 |
| **Post-dose assessment**  (2h after the last dose of ticagrelor) |  |  |  |  |  |  |  |  |
| Ticagrelor (ng/mL) | 703  (517-889) | 1011  (825-1197) | -308  (-510, -105) | 0.004 | 737  (528-945) | 1407  (1198-1615) | -670  (-898, -442) | < 0.001 |
| AR-C124910XX (ng/mL) | 297  (224-370) | 453  (379-526) | -156  (-247, -63.7) | < 0.001 | 334  (252-416) | 578  (496-660) | -244  (-348, -141) | < 0.001 |

Values are least square means (95% CI).

^1^P-value for comparison between treatment.

**eFigure 1**.


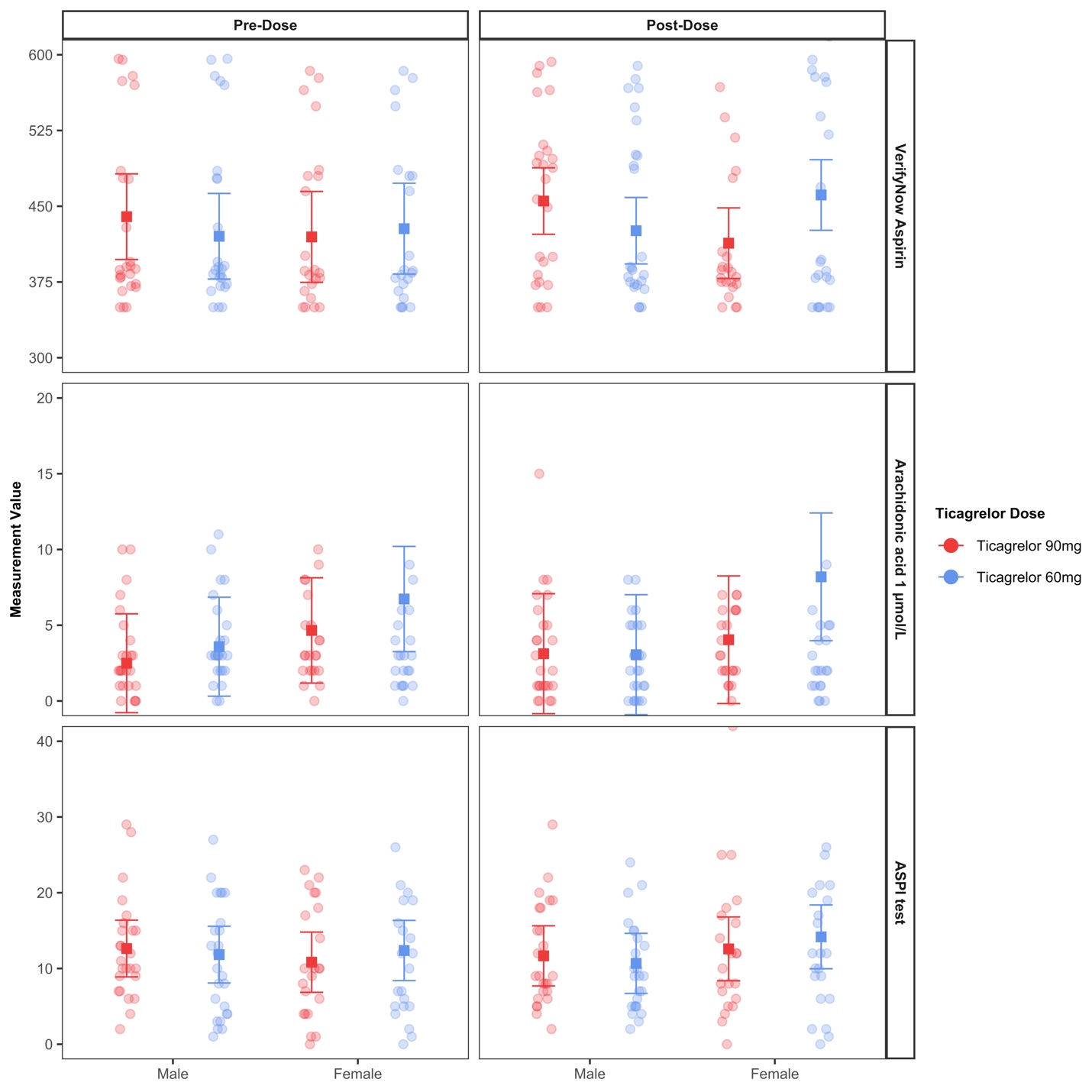

Supplement: Supplementary file 1 — Supplementary Material 1 [file 11239_2025_3206_MOESM1_ESM.docx]
